# Pan-immune inflammation value and system inflammation response index associated with prolonged hospital stay of thyroid nodule patients undergoing ultrasound-guided microwave ablation, both aged ≤55 years and > 55 years

**DOI:** 10.3389/fendo.2026.1786577

**Published:** 2026-04-17

**Authors:** Mingwei Song, Sirong Lan, Hui Rao, Yeqian Lai

**Affiliations:** 1 Department of Ultrasound, Meizhou People’s Hospital, Meizhou Academy of Medical Sciences, Meizhou, China; 2 Department of Laboratory Medicine, Meizhou People’s Hospital, Meizhou Academy of Medical Sciences, Meizhou, China; 3 Department of Thyroid Surgery, Meizhou People’s Hospital, Meizhou Academy of Medical Sciences, Meizhou, China

**Keywords:** microwave ablation, pan-immune inflammation value, prolonged hospital stay, system inflammation response index, thyroid nodule

## Abstract

**Background:**

The preoperative immune inflammatory status may affect the prognosis of thyroid nodule patients performed ultrasound-guided microwave ablation therapy. The aim of this study is to analyze the relationship between the pan-immune inflammation value (PIV), systemic immune inflammation index (SII), and system inflammation response index (SIRI) and the prolonged hospital stay of patients after microwave ablation.

**Methods:**

1288 patients who underwent ultrasound-guided microwave ablation for thyroid nodules from October 2017 to March 2024 were retrospectively analyzed. The patients were divided into prolonged hospital stay group (hospital stay >1 day) and non-prolonged hospital stay group (1 day). Receiver operating characteristic (ROC) curve analysis was performed to assess the diagnostic efficacy of PIV, SII and SIRI in predicting prolonged hospital stay. Logistic regression analysis was applied to examine the relationship of PIV, SII, SIRI with prolonged hospital stay.

**Results:**

The levels of PIV, SII, and SIRI in prolonged hospital stay group were higher than those in non-prolonged hospital stay group. The cut-off value of PIV, SII, and SIRI was 190.56, 541.31, and 0.845 based on ROC curve analysis, respectively. In subjects with age ≤55 years, logistic regression analysis showed that high PIV (odds ratio (OR): 2.119, 95% confidence interval (CI): 1.389-3.232, *p* < 0.001), SII (OR: 1.678, 95% CI: 1.173-2.400, *p* = 0.005) and SIRI level (OR: 1.948, 95% CI: 1.316-2.882, *p* = 0.001) were associated with prolonged hospital stay. In age >55 years old, diabetes mellitus (OR: 1.720, 95% CI: 1.002-2.950, *p* = 0.049), high PIV (OR: 1.961, 95% CI: 1.086-3.541, *p* = 0.026) and SIRI (OR: 1.840, 95% CI: 1.015-3.335, *p* = 0.044) were significantly associated with prolonged hospital stay.

**Conclusions:**

PIV and SIRI are associated with the prolonged hospital stay of thyroid nodule patients undergoing ultrasound-guided microwave ablation, both aged ≤55 years and > 55 years.

## 1 Introduction

Thyroid nodule refers to an abnormal growth of local thyroid cells, resulting in the formation of one or more abnormal tissue masses within the thyroid gland (1). Thyroid nodules can present as cystic, solid, or mixed cystic and solid structures (2). In most cases, they exist in the form of solitary or multiple nodules (3). From a pathological perspective, thyroid nodules can be broadly classified into two categories: benign and malignant. Benign nodules account for more than 85% of all cases (4). They usually grow slowly and have no obvious invasiveness. Some can remain in a stable state for a long time.

The traditional treatment methods for thyroid nodules mainly include surgical treatment, radioactive iodine therapy, and drug therapy (5, 6). Surgical treatment is mainly applicable to malignant nodules or large benign nodules (7). However, it is quite invasive and may cause complications such as recurrent laryngeal nerve injury (8) and hypoparathyroidism (9) after the operation. Radioactive iodine therapy is mainly used for patients with hyperthyroidism and nodules (10). It works by using the beta rays of radioactive iodine to damage the thyroid tissue, but this method has a dose-dependent risk of hypothyroidism (11). The therapeutic effect of drugs varies significantly among individuals. It is only effective for some patients and requires long-term medication, and there is a high risk of recurrence (12, 13).

With the advancement of minimally invasive medical technologies, ultrasound-guided thermal ablation techniques (including microwave ablation, radiofrequency ablation, and laser ablation) have gradually emerged as novel therapeutic approaches for benign thyroid nodules and some low-risk malignant thyroid nodules (14, 15). Among them, ultrasound-guides microwave ablation is the most used in clinical practice in the Asian region due to its advantages such as high energy transfer efficiency, strong controllability of the ablation range, and short operation time (16, 17). Generally, if the surgery proceeds smoothly and the patient has no complications, the hospital stay for thyroid nodule ablation surgery is relatively short. After the ablation operation, close monitoring of vital signs and wound conditions is conducted, and the patient can be discharged within one to two days (18–20). However, for some patients, the hospital stay may be prolonged due to various reasons.

Although microwave ablation for thyroid nodules is minimally invasive, thermal coagulation necrosis of the nodular tissue during the procedure can still elicit local inflammatory responses, which may further activate the systemic inflammatory system via blood circulation (21). At the same time, the preoperative potential chronic inflammatory conditions of the patients (such as combined obesity, diabetes mellitus, and chronic pharyngitis) may also superimpose the surgical-related inflammatory responses, further affecting the postoperative recovery process (22). Pan-immune inflammation value (PIV), systemic immune inflammation index (SII), and system inflammation response index (SIRI) are several comprehensive immune-inflammation indices calculated based on several blood cell count indicators (23–26). At present, there are few studies on the relationship between PIV, SII and SIRI and the prolonged hospital stay of patients with thyroid nodules undergoing ultrasound-guided microwave ablation (27, 28). Given the differences in hospital stay for patients with thyroid nodules undergoing this surgery and the close relationship between systemic inflammation and the prognosis of many diseases, it is of great significance to explore the relationship between these inflammatory indicators and prolonged hospital stay. The purpose of this study was to investigate the association PIV, SII, SIRI with prolonged hospital stay of thyroid nodule patients undergoing ultrasound-guided microwave ablation.

## 2 Materials and methods

### 2.1 Study cohort

This study retrospectively collected the information of patients who underwent ultrasound-guided microwave ablation for thyroid nodules at Meizhou People’s Hospital from October 2017 to March 2024. The research was approved by the Medical Ethics Committee of Meizhou People’s Hospital. All procedures were carried out in strict accordance with the provisions of the Helsinki Declaration.

Inclusion criteria: (1) diagnosed as thyroid nodules through ultrasound examination before surgery, and the postoperative pathology confirmed as benign nodules or low-risk thyroid micropapillary carcinoma; (2) first-time undergoing ultrasound-guided microwave ablation surgery; (3) completed blood routine tests (including neutrophils, lymphocytes, monocytes, and platelet count) before surgery, with complete and traceable data; (4) complete medical records. Exclusion criteria: (1) having other malignant tumors or acute infectious diseases; (2) having coagulation dysfunction or severe dysfunction of organs such as the heart, liver, and kidneys; (3) having received anti-inflammatory treatment or immunomodulatory agent treatment within 3 months before the ablation surgery; (4) having prolonged hospital stay due to non-ablation-related reasons (such as sudden cerebral infarction, acute myocardial infarction) during hospitalization; (5) missing key information in the medical records.

### 2.2 Microwave ablation therapy for thyroid nodules

All patients with thyroid nodules in this study underwent standardized ultrasound−guided percutaneous microwave ablation. The procedure was performed by senior specialists in strict accordance with clinical protocols and aseptic principles. Before surgery, cervical ultrasound was used to accurately evaluate the size, number, and volume of thyroid nodules as well as the total thyroid volume, and the puncture path was planned. Relevant examinations were completed to exclude contraindications to surgery. During the operation, the patient was placed in the supine position with the shoulder elevated to expose the neck. After local infiltration anesthesia, the ablation was performed under real−time ultrasound guidance. Ablation parameters were adjusted according to nodule characteristics, and the patient’s vital signs and complications were monitored throughout the procedure. Postoperatively, ultrasound was repeated to assess ablation efficacy and rule out immediate complications.

### 2.3 Data collection

The patient’s medical records information is extracted through the hospital’s electronic medical record system, including: (1) demographic characteristics: age, gender; (2) clinical characteristics: personal habits (history of smoking, and history of alcohol consumption), underlying diseases (hypertension, and diabetes mellitus), whether having Hashimoto’s thyroiditis; and (3) laboratory indicators: preoperative neutrophil count, lymphocyte count, monocyte count, platelet count. The eighth edition of the American Joint Committee on Cancer (AJCC) have adjusted the age-based classification standard for the prognosis of thyroid cancer to 55 years old (29). Therefore, in this study, patients were stratified into two groups according to age: ≤55 years and >55 years. Hypertension is defined as systolic blood pressure ≥ 140 mmHg or diastolic blood pressure ≥ 90 mmHg (30); Diabetes mellitus is defined as fasting blood glucose ≥ 7.0 mmol/L or glycosylated hemoglobin ≥ 6.5%. Smoking status is defined as having smoked at least one cigarette per day for one year or more, or having quit smoking for less than six months. Alcohol consumption is defined as regular intake of alcoholic beverages at least once weekly for six or more consecutive months.

### 2.4 Data processing

The comprehensive immune-inflammation indices PIV, SII, and SIRI were calculated according to the following formula:


PIV=monocyte×neutrophil×platelet/lymphocyte;

SII=platelet×neutrophil/lymphocyte;

SIRI=monocyte×neutrophil/lymphocyte.

The discharge criteria were generally based on stable vital signs, no obvious perioperative complications, relief of local pain and discomfort, and normal results of routine laboratory examinations. The hospital stay refers to the time interval from the day of admission to the day of discharge. This study divided the subjects into the prolonged hospital stay group (>1 day) and the non-prolonged hospital stay group (1 day) based on the length of hospital stay. The cutoff value of >1 day was used to define prolonged hospital stay. This threshold was selected to identify patients with a length of stay obviously longer than the most common postoperative stay after minimally invasive surgery, and it was adopted based on previous relevant studies (18, 19). However, this definition is relatively stringent, and the optimal cutoff for prolonged hospital stay remains inconsistent across literature (31, 32).

### 2.5 Statistical analysis

All statistical analysis were performed using SPSS statistical software version 26.0 (IBM Inc., USA). Comparisons among continuous variables that follow a normal distribution were analyzed using independent sample t-test or one-way analysis of variance (ANOVA). Comparisons among continuous variables that do not follow a normal distribution are conducted using Mann-Whitney U test for groups comparisons or correlation analysis. The categorical variables were compared using by χ^2^ test. Receiver operating characteristic (ROC) curve analysis was performed to assess the diagnostic efficacy of PIV, SII, and SIRI in predicting prolonged hospital stay. The area under the ROC curve (AUC) was computed to quantify the discriminatory power of these three indicators for prolonged hospital stay, while the optimal cutoff values for PIV, SII, and SIRI were identified via the Youden index. Logistic regression analysis was applied to examine the relationship of PIV, SII, SIRI with prolonged hospital stay of thyroid nodule patients undergoing ultrasound-guided microwave ablation. A value of *p* < 0.05 was considered statistically significant.

## 3 Results

### 3.1 Characteristics of subjects

A total of 1288 patients were collected in this study, of which 300 (23.3%) were males and 988 (76.7%) were females. Of these patients, 43 (3.3%) had a smoking history, 18 (1.4%) had a drinking history, 220 (17.1%) had hypertension, 104 (8.1%) had diabetes mellitus, and 60 (4.7%) had Hashimoto’s thyroiditis. The mean level of PIV, SII, and SIRI was 194.71 (134.94, 275.27), 495.03 (376.74, 668.76), and 0.77 (0.54, 1.08), respectively (**Table 1**).

**Table 1:** Comparison of clinical features in patients with thyroid nodules who undergo microwave ablation treatment.

Variables	Patients (n=1288)
Gender	
Male, n(%)	300 (23.3%)
Female, n(%)	988 (76.7%)
Age (years)	
≤55, n (%)	848 (65.8%)
>55, n (%)	440 (34.2%)
History of smoking	
No, n(%)	1245 (96.7%)
Yes, n(%)	43 (3.3%)
History of alcohol consumption	
No, n(%)	1270 (98.6%)
Yes, n(%)	18 (1.4%)
Hypertension	
No, n(%)	1068 (82.9%)
Yes, n(%)	220 (17.1%)
Diabetes mellitus	
No, n(%)	1184 (91.9%)
Yes, n(%)	104 (8.1%)
Hashimoto's thyroiditis	
No, n (%)	1228 (95.3%)
Yes, n (%)	60 (4.7%)
Inflammatory indices levels	
PIV, mmol/L, median (IQR)	194.71 (134.94, 275.27)
SII, mmol/L, median (IQR)	495.03 (376.74, 668.76)
SIRI, median (IQR)	0.77 (0.54, 1.08)

PIV, pan-immune-inflammation value; SII, systemic immune-inflammatory index; SIRI, systemic inflammatory response index; IQR, interquartile range.

### 3.2 Comparison of clinical features in patients with and without prolonged hospital stay

In this study, 488 (37.9%) patients had prolonged hospital stay and 800 (62.1%) patients did not. The proportion of diabetes mellitus (10.0% vs. 6.9%, *p* = 0.046) in patients with prolonged hospital stay was higher than that in patients without prolonged hospital stay. The levels of PIV (223.73 (162.91, 323.69) vs. 176.25 (122.72, 246.65), *p* < 0.001), SII (506.73 (393.39, 715.00) vs. 493.16 (368.14, 650.58), *p* = 0.004), and SIRI (0.89 (0.64, 1.23) vs. 0.70 (0.50, 0.97), *p* < 0.001) in patients with prolonged hospital stay were significantly higher than those in patients without prolonged hospital stay. There was no statistically significant difference in gender and age distributions and proportions of smoking, drinking, hypertension, and Hashimoto’s thyroiditis between the two groups (**Table 2**).

**Table 2:** Comparison of clinical features in patients with prolonged hospital stay and without prolonged hospital stay.

Variables	Without prolonged hospital stay (n=800)	With prolonged hospital stay (n=488)	*P (*χ^2^/Z)
Gender			
Male, n(%)	182 (22.8%)	118 (24.2%)	0.587 (χ^2^ = 0.347)
Female, n(%)	618 (77.3%)	370 (75.8%)	
Age (years)			
≤55, n (%)	519 (64.9%)	329 (67.4%)	0.364 (χ^2^ = 0.872)
>55, n (%)	281 (35.1%)	159 (32.6%)	
History of smoking			
No, n(%)	779 (97.4%)	466 (95.5%)	0.079 (χ^2^ = 3.331)
Yes, n(%)	21 (2.6%)	22 (4.5%)	
History of alcohol consumption			
No, n(%)	791 (98.9%)	479 (98.2%)	0.331 (χ^2^ = 1.138)
Yes, n(%)	9 (1.1%)	9 (1.8%)	
Hypertension			
No, n(%)	672 (84.0%)	396 (81.1%)	0.195 (χ^2^ = 1.741)
Yes, n(%)	128 (16.0%)	92 (18.9%)	
Diabetes mellitus			
No, n(%)	745 (93.1%)	439 (90.0%)	0.046 (χ^2^ = 4.093)
Yes, n(%)	55 (6.9%)	49 (10.0%)	
Hashimoto's thyroiditis			
No, n (%)	760 (95.0%)	468 (95.9%)	0.498 (χ^2^ = 0.555)
Yes, n (%)	40 (5.0%)	20 (4.1%)	
Inflammatory indices levels			
PIV, mmol/L, median (IQR)	176.25 (122.72, 246.65)	223.73 (162.91, 323.69)	<0.001 (Z=-8.525)
SII, mmol/L, median (IQR)	493.16 (368.14, 650.58)	506.73 (393.39, 715.00)	0.004 (Z=-2.844)
SIRI, median (IQR)	0.70 (0.50, 0.97)	0.89 (0.64, 1.23)	<0.001 (Z=-8.847)

PIV, pan-immune-inflammation value; SII, systemic immune-inflammatory index; SIRI, systemic inflammatory response index; IQR, interquartile range.

### 3.3 Comparison of clinical features in patients with and without prolonged hospital stay in subjects with age ≤55 years old and >55 years old, respectively

In subjects with age ≤55 years old, the proportions of male (20.1% vs. 14.5%, *p* = 0.037), and history of smoking (4.0% vs. 1.5%, *p* = 0.039) in patients with prolonged hospital stay were higher than those in patients without prolonged hospital stay. The levels of PIV (224.00 (167.78, 324.21) vs. 174.75 (120.98, 250.19), *p* < 0.001), SII (510.49 (410.77, 715.00) vs. 495.57 (363.76, 666.32), *p* = 0.004), and SIRI (0.88 (0.65, 1.20) vs. 0.67 (0.49, 0.94), *p* < 0.001) in patients with prolonged hospital stay were significantly higher than those in patients without prolonged hospital stay (**Table 3**).

**Table 3:** Comparison of clinical features in patients with prolonged hospital stay and without prolonged hospital stay in subjects with age ≤55 years old and >55 years old, respectively.

Variables	Age ≤55 years old (n=848)			Age >55 years old (n=440)		
	Without prolonged hospital stay (n=519)	With prolonged hospital stay (n=329)	*p (*χ^2^/Z)	Without prolonged hospital stay (n=281)	With prolonged hospital stay (n=159)	*P (*χ^2^/Z)
Gender						
Male, n(%)	75 (14.5%)	66 (20.1%)	0.037 (χ^2^ = 4.571)	107 (38.1%)	52 (32.7%)	0.302 (χ^2^ = 1.271)
Female, n(%)	444 (85.5%)	263 (79.9%)		174 (61.9%)	107 (67.3%)	
History of smoking						
No, n(%)	511 (98.5%)	316 (96.0%)	0.039 (χ^2^ = 4.842)	268 (95.4%)	150 (94.3%)	0.653 (χ^2^ = 0.229)
Yes, n(%)	8 (1.5%)	13 (4.0%)		13 (4.6%)	9 (5.7%)	
History of alcohol consumption						
No, n(%)	514 (99.0%)	323 (98.2%)	0.353 (χ^2^ = 1.164)	277 (98.6%)	156 (98.1%)	1.000 (χ^2^ = 0.139)
Yes, n(%)	5 (1.0%)	6 (1.8%)		4 (1.4%)	3 (1.9%)	
Hypertension						
No, n(%)	475 (91.5%)	297 (90.3%)	0.540 (χ^2^ = 0.385)	197 (70.1%)	99 (62.3%)	0.112 (χ^2^ = 2.837)
Yes, n(%)	44 (8.5%)	32 (9.7%)		84 (29.9%)	60 (37.7%)	
Diabetes mellitus						
No, n(%)	501 (96.5%)	313 (95.1%)	0.370 (χ^2^ = 1.018)	244 (86.8%)	126 (79.2%)	0.042 (χ^2^ = 4.370)
Yes, n(%)	18 (3.5%)	16 (4.9%)		37 (13.2%)	33 (20.8%)	
Hashimoto's thyroiditis						
No, n (%)	489 (94.2%)	315 (95.7%)	0.346 (χ^2^ = 0.952)	271 (96.4%)	153 (96.2%)	1.000 (χ^2^ = 0.013)
Yes, n (%)	30 (5.8%)	14 (4.3%)		10 (3.6%)	6 (3.8%)	
Inflammatory indices levels						
PIV, mmol/L, median (IQR)	174.75 (120.98, 250.19)	224.00 (167.78, 324.21)	<0.001 (Z=-7.510)	180.08 (127.97, 241.65)	221.12 (153.56, 322.89)	<0.001 (Z=-4.115)
SII, mmol/L, median (IQR)	495.57 (363.76, 666.32)	510.49 (410.77, 715.00)	0.004 (Z=-2.853)	490.33 (374.51, 618.05)	493.94 (367.36, 717.84)	0.451 (Z=-0.754)
SIRI, median (IQR)	0.67 (0.49, 0.94)	0.88 (0.65, 1.20)	<0.001 (Z=-8.332)	0.76 (0.53, 1.05)	0.90 (0.62, 1.34)	<0.001 (Z=-3.727)

PIV, pan-immune-inflammation value; SII, systemic immune-inflammatory index; SIRI, systemic inflammatory response index; IQR, interquartile range.

In patients with age >55 years old, the proportions of diabetes mellitus (20.8% vs. 13.2%, *p* = 0.042) in prolonged hospital stay group was higher than that in non-prolonged hospital stay group. The levels of PIV (221.12 (153.56, 322.89) vs. 180.08 (127.97, 241.65), *p* < 0.001), and SIRI (0.90 (0.62, 1.34) vs. 0.76 (0.53, 1.05), *p* < 0.001) in patients with prolonged hospital stay were significantly higher than those in patients without prolonged hospital stay (**Table 3**).

### 3.4 Logistic regression analysis of associated factors with prolonged hospital stay in patients with thyroid nodules who undergo microwave ablation treatment

**Figure 1:**
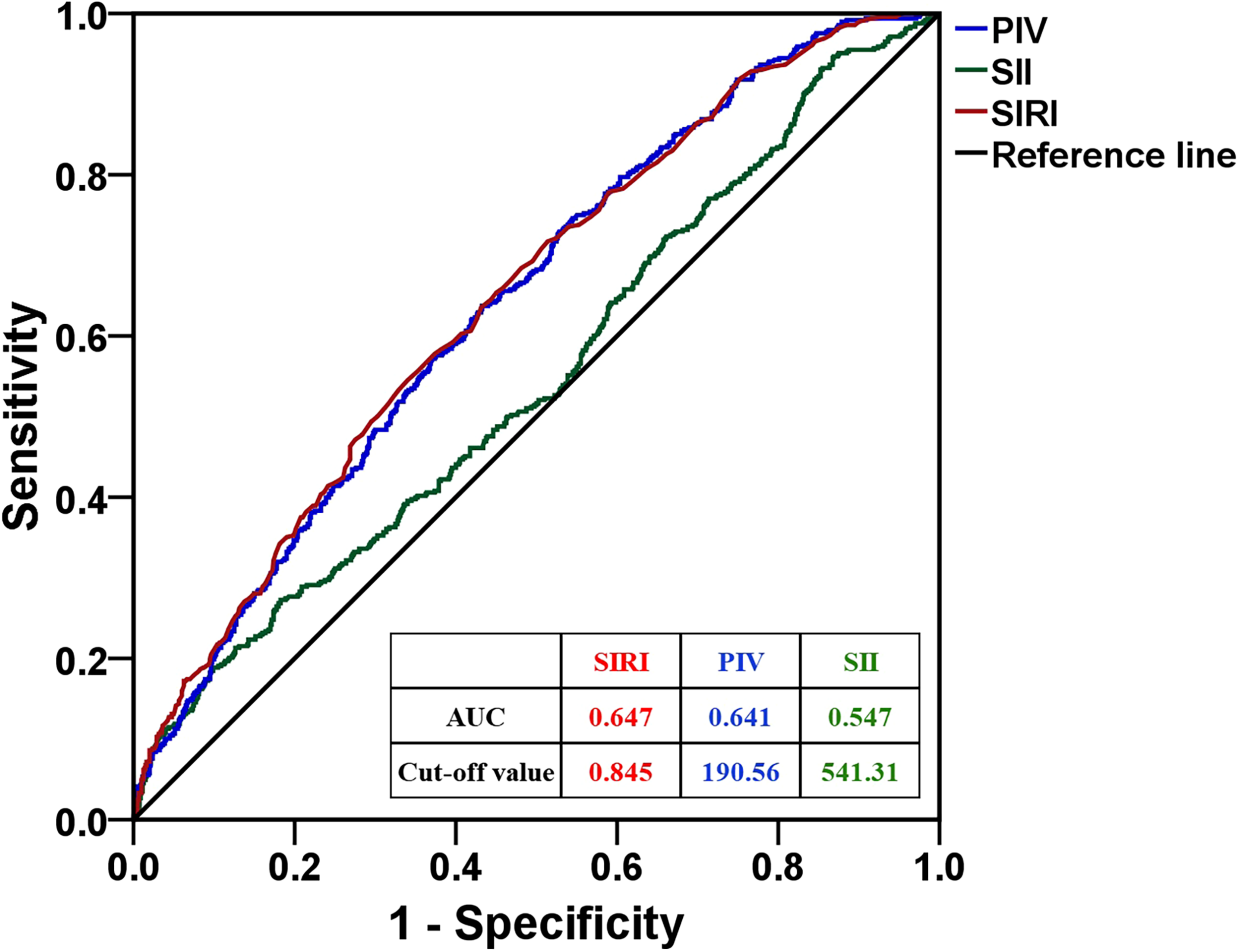
The ROC curve analysis of PIV, SII, and SIRI to distinguish prolonged hospital stay. PIV, pan-immune-inflammation value; SII, systemic immune-inflammatory index; SIRI, systemic inflammatory response index.

When prolonged hospital stay was considered as the endpoint of PIV, SII, and SIRI levels in ROC curve analysis, the cut-off value of PIV, SII, and SIRI was 190.56, 541.31, and 0.845, respectively (**Figure 1**). The results of univariate logistic regression analysis showed that diabetes mellitus (odds ratio (OR): 1.512, 95% confidence interval (CI): 1.011-2.262, *p* = 0.044), high PIV level (≥190.56 vs. <190.56, OR: 2.306, 95% CI: 1.829-2.906, *p* < 0.001) and SIRI level (≥0.845 vs. <0.845, OR: 2.326, 95% CI: 1.847-2.929, *p* < 0.001) were significantly associated with prolonged hospital stay. Multivariate logistic regression analysis showed that high PIV level (≥190.56 vs. <190.56, OR: 2.052, 95% CI: 1.459-2.886, *p* < 0.001), SII level (≥541.31 vs. <541.31, OR: 1.607, 95% CI: 1.211-2.133, *p* = 0.001), and SIRI level (≥0.845 vs. <0.845, OR: 1.854, 95% CI: 1.340-2.566, *p* < 0.001) were significantly associated with prolonged hospital stay (**Table 4**).

**Table 4:** Logistic regression analysis of associated factors with prolonged hospital stay in patients with thyroid nodules who undergo microwave ablation treatment.

Variables	Univariate OR (95% CI)	*p* values	Multivariate OR (95% CI)	*P* values
Gender (female vs. male)	0.923 (0.708-1.204)	0.556	1.429 (1.044-1.955)	0.026
Age (>55 vs. ≤55 years old)	0.893 (0.703-1.133)	0.351	0.813 (0.621-1.065)	0.133
History of smoking (yes vs. no)	1.751 (0.953-3.219)	0.071	1.741 (0.845-3.586)	0.132
History of alcoholism (yes vs. no)	1.651 (0.651-4.189)	0.291	1.419 (0.487-4.129)	0.521
Hypertension (yes vs. no)	1.220 (0.908-1.639)	0.187	1.280 (0.924-1.774)	0.137
Diabetes mellitus (yes vs. no)	1.512 (1.011-2.262)	0.044	1.509 (0.980-2.323)	0.062
Hashimoto's thyroiditis (yes vs. no)	0.812 (0.469-1.406)	0.457	0.712 (0.401-1.265)	0.247
PIV (≥190.56 vs. <190.56)	2.306 (1.829-2.906)	<0.001	2.052 (1.459-2.886)	<0.001
SII (≥541.31 vs. <541.31)	1.181 (0.941-1.484)	0.152	1.607 (1.211-2.133)	0.001
SIRI (≥0.845 vs. <0.845)	2.326 (1.847-2.929)	<0.001	1.854 (1.340-2.566)	<0.001

OR, odds ratio; CI, confidence interval; PIV, pan-immune-inflammation value; SII, systemic immune-inflammatory index; SIRI, systemic inflammatory response index.

### 3.5 Logistic regression analysis of associated factors with prolonged hospital stay in patients with ≤55 years old and >55 years old, respectively

In subjects with age ≤55 years old, multivariate logistic regression analysis showed that high PIV level (≥190.56 vs. <190.56, OR: 2.119, 95% CI: 1.389-3.232, *p* < 0.001), SII level (≥541.31 vs. <541.31, OR: 1.678, 95% CI: 1.173-2.400, *p* = 0.005), and SIRI level (≥0.845 vs. <0.845, OR: 1.948, 95% CI: 1.316-2.882, *p* = 0.001) were significantly associated with prolonged hospital stay (**Table 5**).

**Table 5:** Logistic regression analysis of associated factors with prolonged hospital stay in patients with ≤55 years old and >55 years old, respectively.

Variables	Age ≤55 years old				Age >55 years old			
	Unadjusted values		Adjusted values		Unadjusted values		Adjusted values	
	OR (95% CI)	*P* values	Adjusted OR (95% CI)	*P* values	OR (95% CI)	*P* values	Adjusted OR (95% CI)	*P* values
Gender (female vs. male)	0.673(0.468-0.969)	0.033	1.011(0.672-1.523)	0.957	1.265(0.840-1.906)	0.260	2.209(1.332-3.664)	0.002
History of smoking (yes vs. no)	2.628(1.077-6.411)	0.034	1.810(0.642-5.103)	0.262	1.237(0.517-2.962)	0.633	1.742(0.598-5.078)	0.309
History of alcoholism (yes vs. no)	1.910(0.578-6.308)	0.289	1.342(0.343-5.257)	0.673	1.332(0.294-6.027)	0.710	1.434(0.243-8.462)	0.690
Hypertension (yes vs. no)	1.163(0.721-1.876)	0.535	1.167(0.707-1.926)	0.547	1.421(0.943-2.142)	0.093	1.350(0.875-2.083)	0.174
Diabetes mellitus (yes vs. no)	1.423(0.715-2.831)	0.315	1.182(0.575-2.432)	0.649	1.727(1.031-2.894)	0.038	1.720(1.002-2.950)	0.049
Hashimoto's thyroiditis (yes vs. no)	0.724(0.378-1.388)	0.331	0.630(0.318-1.248)	0.185	1.063(0.379-2.981)	0.908	0.953(0.321-2.824)	0.930
PIV (≥190.56 vs. <190.56)	2.470(1.856-3.286)	<0.001	2.119(1.389-3.232)	<0.001	2.010(1.352-2.987)	0.001	1.961(1.086-3.541)	0.026
SII (≥541.31 vs. <541.31)	1.194(0.904-1.578)	0.212	1.678(1.173-2.400)	0.005	1.142(0.767-1.699)	0.514	1.440(0.894-2.319)	0.134
SIRI (≥0.845 vs. <0.845)	2.601(1.956-3.458)	<0.001	1.948(1.316-2.882)	0.001	1.896(1.279-2.812)	0.001	1.840(1.015-3.335)	0.044

OR, odds ratio; CI, confidence interval; PIV, pan-immune-inflammation value; SII, systemic immune-inflammatory index; SIRI, systemic inflammatory response index.

In subjects with age >55 years old, multivariate logistic regression analysis showed that diabetes mellitus (OR: 1.720, 95% CI: 1.002-2.950, *p* = 0.049), high PIV level (≥190.56 vs. <190.56, OR: 1.961, 95% CI: 1.086-3.541, *p* = 0.026), and SIRI level (≥0.845 vs. <0.845, OR: 1.840, 95% CI: 1.015-3.335, *p* = 0.044) were significantly associated with prolonged hospital stay (**Table 5**).

## 4 Discussion

This study conducted a retrospective analysis of data from 1288 patients who underwent ultrasound-guided microwave ablation for thyroid nodules. It was the first to systematically explore the association between preoperative PIV, SII, and SIRI and prolonged hospital stay (>1 day). The results showed that PIV and SIRI are factors influencing the prolonged hospital stay of thyroid nodule patients undergoing ultrasound-guided microwave ablation, both aged ≤55 years and > 55 years. This finding not only confirmed the crucial impact of preoperative systemic inflammatory status on the recovery process after minimally invasive thyroid ablation, but also provided a set of convenient and low-cost prediction tools based on routine blood tests, providing direct evidence support for the clinical implementation of “preoperative risk stratification - individualized intervention”.

PIV and SIRI reflect the status of the pro-inflammatory - anti-inflammatory balance through the ratio of neutrophils, monocytes and lymphocytes (33). An increase in neutrophils indicates that the body is in an acute inflammatory activation state (34). The neutrophil elastase and oxygen free radicals released by them can aggravate the tissue edema (35) and oxidative stress damage (36, 37) in the ablated area, delaying local repair. Monocytes, as anti-inflammatory and phagocytic cells, although they can remove necrotic tissues, excessive activation will differentiate into macrophages and secrete inflammatory mediators such as interleukin 6 (IL-6) and tumor necrosis factor-alpha (TNF-α), forming an “inflammation persistence” effect (38), increasing the risk of postoperative hematoma or minor infection, and thereby prolonging the observation period (39, 40). Lymphocyte reduction indicates the inhibition of cellular immune function. Postoperative tissue repair relies on the immune regulation mediated by T lymphocytes (41, 42). A decrease in lymphocyte count will lead to a decline in the repair efficiency, thereby indirectly prolonging the hospital stay. Platelets are not only crucial components for blood clotting, but they can also release mediators such as platelet-derived growth factor (PDGF) and platelet factor 4 (PF4), which can promote the chemotaxis and adhesion of neutrophils, further intensifying the local inflammatory response (43, 44). At the same time, the microthrombi formed after platelet activation may block the tiny blood vessels in the ablation area, affecting local blood supply and delaying the absorption of necrotic tissues (45).

Lu et al. found that high PIV was a risk factor of prolonged length of hospital stay in patients with acute exacerbation of chronic obstructive pulmonary disease (AECOPD) (46). The research results of Cai et al. suggested that PIV might be an independent predictor for prolonged hospital stay, increased overall and major postoperative complication rates, and decreased long-term survival rate in patients with acute type A aortic dissection (TAAD) (47). In patients with pneumonia complicated by respiratory failure who received blood transfusion treatment, a high SII was associated with prolonged intensive care unit (ICU) stay (48).

From the perspective of age stratification, elderly patients (aged > 55 years) have declined physiological functions and weakened immune and inflammatory regulation capabilities (49). The preoperative immune-inflammatory indices (PIV, SIRI) of elderly patients may be at a relatively high level. After microwave ablation surgery, the inflammatory response is more intense and lasts longer, making it difficult for them to quickly return to normal. Therefore, it is prone to significantly prolong the hospital stay. While younger patients (aged ≤ 55 years) have relatively better physical functions and stronger immune and inflammatory regulation capabilities, they still have a stronger ability to regulate and recover from the inflammatory response (50). However, high PIV and SIRI still have adverse effects on the hospital stay. This result indicates that the impact of inflammatory response on postoperative recovery cannot be ignored in all age groups.

Although this study provides valuable evidence for clinical practice, it still has the following limitations that need to be addressed in future research. Firstly, this study was a single-center retrospective investigation, which may be subjected to selection bias and limit the generalizability and external validity of the results to a certain extent. Therefore, large-sample, prospective, and multi-center studies with longer follow-up periods are warranted in the future to further verify, supplement, and improve the preliminary conclusions of this study. Secondly, this study was designed as a retrospective observational study, and the characteristics of thyroid nodules (such as nodule size and nodule structure), some detailed perioperative parameters (such as the duration of surgery, hematoma, and local pain), and some detailed reasons for prolonged hospitalization were not included in the present study. Finally, this study only inferred the mechanism of the inflammatory index based on clinical data, and did not verify the causal relationship of “increased inflammatory indicators→aggravated inflammation in the ablated area→prolonged hospital stay” through animal experiments or cell experiments. In the future, combined with basic experiments, we can further explore the molecular mechanism of inflammatory cells (such as neutrophils) and thyroid tissue repair, providing more precise targets for clinical intervention. And prospective studies with standardized data collection, detailed recording of operation time, and systematic evaluation of perioperative complications are warranted to verify and expand our findings.

## 5 Conclusion

This study confirms that preoperative PIV and SIRI are associated with prolonged hospital stay following ultrasound−guided microwave ablation for thyroid nodules, regardless of age (≤55 or >55 years). As readily available biomarkers derived from routine laboratory tests, PIV and SIRI are simple, cost-effective, and useful for preoperative risk stratification. They can help clinicians develop individualized treatment strategies, shorten hospital stays, and optimize medical resource allocation. Further prospective multicenter studies are warranted to validate these findings and explore preoperative interventions (e.g., anti−inflammatory therapy) targeting these indices to improve outcomes in patients undergoing thyroid ablation.

## Data Availability

The original contributions presented in the study are included in the article/supplementary material. Further inquiries can be directed to the corresponding author.

## References

[1] Alexander EK Cibas ES . Diagnosis of thyroid nodules. Lancet Diabetes Endocrinol. (2022) 10:533–9. doi: 10.1016/S2213-8587(22)00101-2. PMID: 35752200

[2] Teefey SA Middleton WD Reading CC Langer JE Beland MD Szabunio MM . Effect of decreasing the ACR TI-RADS point assignment for punctate echogenic foci when they occur in mixed solid and cystic thyroid nodules. AJR Am J Roentgenol. (2021) 216:479–85. doi: 10.2214/AJR.20.22793. PMID: 33295817

[3] Rehman AU Ehsan M Javed H Ameer MZ Mohsin A Aemaz Ur Rehman M . Solitary and multiple thyroid nodules as predictors of Malignancy: a systematic review and meta-analysis. Thyroid Res. (2022) 15:22. doi: 10.1186/s13044-022-00140-6. PMID: 36464691 PMC9720983

[4] Wang J Du J Tao C Qi M Yan J Hu B . Classification of benign-malignant thyroid nodules based on hyperspectral technology. Sensors (Basel). (2024) 24:3197. doi: 10.3390/s24103197. PMID: 38794051 PMC11126106

[5] Abraham PJ Lindeman BM . Management of incidental thyroid nodules. Surg Clin North Am. (2024) 104:711–23. doi: 10.1016/j.suc.2024.02.002. PMID: 38944493

[6] Uludag M Unlu MT Kostek M Aygun N Caliskan O Ozel A . Management of thyroid nodules. Sisli Etfal Hastan Tip Bul. (2023) 57:287–304. doi: 10.14744/SEMB.2023.06992. PMID: 37900341 PMC10600596

[7] Durante C Hegedüs L . 2023 European Thyroid Association Clinical Practice Guidelines for thyroid nodule management. Eur Thyroid J. (2023) 12:e230067. doi: 10.1530/ETJ-23-0067. PMID: 37358008 PMC10448590

[8] Gunn A Oyekunle T Stang M Kazaure H Scheri R . Recurrent laryngeal nerve injury after thyroid surgery: an analysis of 11,370 patients. J Surg Res. (2020) 255:42–9. doi: 10.1016/j.jss.2020.05.017. PMID: 32540579

[9] Yazıcıoğlu M Yılmaz A . Risks and prediction of postoperative hypoparathyroidism due to thyroid surgery. Sci Rep. (2021) 11:11876. doi: 10.1038/s41598-021-91277-1. PMID: 34088943 PMC8178369

[10] Yoshihara A Yoshimura Noh J Inoue K . Long-term outcomes after a radioactive iodine treatment for a single autonomous functioning thyroid nodule in Japan. Endocr J. (2025) 72:487–94. doi: 10.1507/endocrj.EJ24-0578. PMID: 39842794 PMC12086274

[11] Varghese J Rohren E Guofan X . Radioiodine imaging and treatment in thyroid disorders. Neuroimaging Clin N Am. (2021) 31:337–44. doi: 10.1016/j.nic.2021.04.003. PMID: 34243868

[12] Soto Jacome C Arce-Camposano A Toro-Tobon D . Drug repurposing for reducing the size of benign thyroid nodules: a systematic review. J Clin Endocrinol Metab. (2026) 111:e327–38. doi: 10.1210/clinem/dgaf616. PMID: 41225692 PMC12819850

[13] Ouyang S Li W Yu P Li H Cai H Wu J . Effect of Chinese herbal medicine for patients with benign thyroid nodules in adults: a protocol for systematic review and meta-analysis. Medicine. (2021) 100:e24591. doi: 10.1097/MD.0000000000024591. PMID: 33663069 PMC7909165

[14] Qian Y Li Z Fan C Huang Y . Comparison of ultrasound-guided microwave ablation, laser ablation, and radiofrequency ablation for the treatment of elderly patients with benign thyroid nodules: a meta-analysis. Exp Gerontol. (2024) 191:112425. doi: 10.1016/j.exger.2024.112425. PMID: 38604254

[15] Russotto F Fiorentino V . Histologic evaluation of thyroid nodules treated with thermal ablation: an institutional experience. Int J Mol Sci. (2024) 25:10182. doi: 10.3390/ijms251810182. PMID: 39337667 PMC11432105

[16] Erturk MS Cekic B Celik M Ucar H . Microwave ablation of symptomatic benign thyroid nodules: short- and long-term effects on thyroid function tests, thyroglobulin and thyroid autoantibodies. Clin Endocrinol (Oxf). (2021) 94:677–83. doi: 10.1111/cen.14348. PMID: 33020965

[17] Turkoglu S Yilmaz AH Yokus A Ulutaş ME . Comparison of microwave ablation and lobectomy in the treatment of benign thyroid nodules. J Clin Ultrasound. (2025) 53:1713–20. doi: 10.1002/jcu.24071. PMID: 40351191

[18] Min X Zhang Z Chen Y Zhao S Ge J Zhao H . Comparison of the effectiveness of lauromacrogol injection for ablation and microwave ablation in the treatment of predominantly cystic thyroid nodules: a multicentre study. BMC Cancer. (2023) 23:785. doi: 10.1186/s12885-023-11301-7. PMID: 37612615 PMC10464182

[19] Yang H Chen Y Chen B Zhao S Zhang Z Wang K . Ablating aspiration needle tract prior to microwave ablation can improve therapeutic outcomes for predominantly cystic thyroid nodules. Front Endocrinol. (2021) 12:752822. doi: 10.3389/fendo.2021.752822. PMID: 34630337 PMC8498334

[20] Zhang P Wang L Li G Wei T Zhu J Lei J . Psychological impacts of thermal ablation and conventional thyroidectomy in BTN patients: a prospective observational study. Endocrine. (2024) 85:1310–8. doi: 10.1007/s12020-024-03814-3. PMID: 38598064

[21] Erturk MS Cekic B Celik M . Microwave ablation of benign thyroid nodules: effects on systemic inflammatory response. J Coll Physicians Surg Pak. (2020) 30:694–700. doi: 10.29271/jcpsp.2020.07.694. PMID: 32811597

[22] Dou JP Yu J Cheng ZG Liu FY Yu XL Hou QD . Symptomatic aseptic necrosis of benign thyroid lesions after microwave ablation: risk factors and clinical significance. Int J Hyperthermia. (2021) 38:815–22. doi: 10.1080/02656736.2021.1930203. PMID: 34039239

[23] Yang XC Liu H Liu DC Tong C Liang XW Chen RH . Prognostic value of pan-immune-inflammation value in colorectal cancer patients: a systematic review and meta-analysis. Front Oncol. (2022) 12:1036890. doi: 10.3389/fonc.2022.1036890. PMID: 36620576 PMC9813847

[24] Xia Y Xia C Wu L . Systemic immune inflammation index (SII), system inflammation response index (SIRI) and risk of all-cause mortality and cardiovascular mortality: a 20-year follow-up cohort study of 42,875 US adults. J Clin Med. (2023) 12:1128. doi: 10.3390/jcm12031128. PMID: 36769776 PMC9918056

[25] Ye C Yuan L Wu K Shen B Zhu C . Association between systemic immune-inflammation index and chronic obstructive pulmonary disease: a population-based study. BMC Pulm Med. (2023) 23:295. doi: 10.1186/s12890-023-02583-5. PMID: 37563621 PMC10416535

[26] Çakır N Koc AN . Gamma-glutamyl transpeptidase-platelet ratio, systemic immune inflammation index, and system inflammation response index in invasive aspergillosis. Rev Assoc Med Bras. (2021) 67:1021–5. doi: 10.1590/1806-9282.20210475. PMID: 34817517

[27] Refaat L Eissa MS Elnaggar GN Mehesen M Eissa MS Kamal A . Systemic inflammatory indices comprising monocytes provide a clinical significance for thyroid cancer identification. Sci Rep. (2025) 15:39967. doi: 10.1038/s41598-025-23765-7. PMID: 41238711 PMC12618645

[28] Çatak M Koca B Çetin Z Başer Ö . Evaluation of three inflammation-associated blood indices for predicting Malignancy in thyroid nodules. Arch Endocrinol Metab. (2026) 70:e260014. doi: 10.20945/2359-4292-2026-0014. PMID: 41746152 PMC12944293

[29] Zanoni DK Patel SG Shah JP . Changes in the 8th edition of the American Joint Committee on Cancer (AJCC) staging of head and neck cancer: rationale and implications. Curr Oncol Rep. (2019) 21:52. doi: 10.1007/s11912-019-0799-x. PMID: 30997577 PMC6528815

[30] Wang Z Chen Z Zhang L Wang X Hao G Zhang Z . Status of hypertension in China: results from the China Hypertension Survey, 2012-2015. Circulation. (2018) 137:2344–56. doi: 10.1161/CIRCULATIONAHA.117.032380. PMID: 29449338

[31] de Vries LH Aykan D Lodewijk L Damen JAA Borel Rinkes IHM Vriens MR . Outcomes of minimally invasive thyroid surgery - a systematic review and meta-analysis. Front Endocrinol. (2021) 12:719397. doi: 10.3389/fendo.2021.719397. PMID: 34456874 PMC8387875

[32] Ding J Wang D Zhang W Xu D Wang W . Ultrasound-guided radiofrequency and microwave ablation for the management of patients with benign thyroid nodules: systematic review and meta-analysis. Ultrasound Q. (2023) 39:61–8. doi: 10.1097/RUQ.0000000000000636. PMID: 36763842

[33] Suwadi A Tandarto K Laksono S . Systemic immune-inflammation index as a potential biomarker for predicting acute pulmonary embolism: a systematic review. Rom J Intern Med. (2024) 62:231–40. doi: 10.2478/rjim-2024-0016. PMID: 38595041

[34] Herre M Cedervall J . Neutrophil extracellular traps in the pathology of cancer and other inflammatory diseases. Physiol Rev. (2023) 103:277–312. doi: 10.1152/physrev.00062.2021. PMID: 35951483 PMC9576172

[35] Yamashiro S Arakaki R Kise Y Kuniyoshi Y . Prevention of pulmonary edema after minimally invasive cardiac surgery with mini-thoracotomy using neutrophil elastase inhibitor. Ann Thorac Cardiovasc Surg. (2018) 24:32–9. doi: 10.5761/atcs.oa.17-00102. PMID: 29118307 PMC5833138

[36] Singhal A Kumar S . Neutrophil and remnant clearance in immunity and inflammation. Immunology. (2022) 165:22–43. doi: 10.1111/imm.13423. PMID: 34704249

[37] Lee HT Lin CS Liu CY . Mitochondrial plasticity and glucose metabolic alterations in human cancer under oxidative stress-from viewpoints of chronic inflammation and neutrophil extracellular traps (NETs). Int J Mol Sci. (2024) 25:9458. doi: 10.3390/ijms25179458. PMID: 39273403 PMC11395599

[38] Chimen M Yates CM . Monocyte subsets coregulate inflammatory responses by integrated signaling through TNF and IL-6 at the endothelial cell interface. J Immunol. (2017) 198:2834–43. doi: 10.4049/jimmunol.1601281. PMID: 28193827 PMC5357784

[39] Guzmán-Beltrán S Herrera MT Torres M Gonzalez Y . CD33 is downregulated by influenza virus H1N1pdm09 and induces ROS and the TNF-α, IL-1β, and IL-6 cytokines in human mononuclear cells. Braz J Microbiol. (2022) 53:89–97. doi: 10.1007/s42770-021-00663-4. PMID: 35075617 PMC8882749

[40] Cabrera-Rivera GL Madera-Sandoval RL León-Pedroza JI Ferat-Osorio E Salazar-Rios E . Increased TNF-α production in response to IL-6 in patients with systemic inflammation without infection. Clin Exp Immunol. (2022) 209:225–35. doi: 10.1093/cei/uxac055. PMID: 35647912 PMC9390847

[41] Muñoz-Rojas AR Mathis D . Tissue regulatory T cells: regulatory chameleons. Nat Rev Immunol. (2021) 21:597–611. doi: 10.1038/s41577-021-00519-w. PMID: 33772242 PMC8403160

[42] Zhang M Zhang S . T cells in fibrosis and fibrotic diseases. Front Immunol. (2020) 11:1142. doi: 10.3389/fimmu.2020.01142. PMID: 32676074 PMC7333347

[43] Urbán-Solano A Flores-Gonzalez J Cruz-Lagunas A Pérez-Rubio G Buendia-Roldan I Ramón-Luing LA . High levels of PF4, VEGF-A, and classical monocytes correlate with the platelets count and inflammation during active tuberculosis. Front Immunol. (2022) 13:1016472. doi: 10.3389/fimmu.2022.1016472. PMID: 36325331 PMC9618821

[44] Liu Y Zhang Y Zhang J Ma J Bian K Wang Y . CD226 is required to maintain megakaryocytes/platelets homeostasis in the treatment of knee osteoarthritis with platelet-rich plasma in mice. Front Pharmacol. (2021) 12:732453. doi: 10.3389/fphar.2021.732453. PMID: 34526904 PMC8436152

[45] Wienkamp AK Erpenbeck L Rossaint J . Platelets in the networks interweaving inflammation and thrombosis. Front Immunol. (2022) 13:953129. doi: 10.3389/fimmu.2022.953129. PMID: 35979369 PMC9376363

[46] Lu Y Guo H Zhang Q . High pan-immune inflammation values are associated with prolonged length of hospital stay in patients with acute exacerbation of chronic obstructive pulmonary disease. Int J Gen Med. (2025) 18:4825–35. doi: 10.2147/IJGM.S542944. PMID: 40901373 PMC12399850

[47] Cai H Shourav FM . Impact of pan-immune inflammation value on short-term outcomes and long-term prognosis in patients with type A aortic dissection. J Inflammation Res. (2025) 18:7855–66. doi: 10.2147/JIR.S522998. PMID: 40546410 PMC12182077

[48] Zheng Z Yu M Peng G Xiao Y . Systemic immune inflammation index (SII) and prognostic nutritional index (PNI) associated with prolonged intensive care unit (ICU) stay in patients with pneumonia complicated with respiratory failure. Int J Gen Med. (2025) 18:1765–76. doi: 10.2147/IJGM.S510659. PMID: 40177424 PMC11963809

[49] Dong H Hu F Hao B Jin X Zheng Q Su Y . Single-cell analysis reveals the disparities in immune profiles between younger and elder patients. Eur Geriatr Med. (2024) 15:1509–22. doi: 10.1007/s41999-024-01032-8. PMID: 39244673

[50] Hornsby E Johnston LM . Effect of Pilates intervention on physical function of children and youth: a systematic review. Arch Phys Med Rehabil. (2020) 101:317–28. doi: 10.1016/j.apmr.2019.05.023. PMID: 31152703

